# An accurate and rapidly calibrating speech neuroprosthesis

**DOI:** 10.1101/2023.12.26.23300110

**Published:** 2024-04-10

**Authors:** Nicholas S. Card, Maitreyee Wairagkar, Carrina Iacobacci, Xianda Hou, Tyler Singer-Clark, Francis R. Willett, Erin M. Kunz, Chaofei Fan, Maryam Vahdati Nia, Darrel R. Deo, Aparna Srinivasan, Eun Young Choi, Matthew F. Glasser, Leigh R. Hochberg, Jaimie M. Henderson, Kiarash Shahlaie, David M. Brandman, Sergey D. Stavisky

**Affiliations:** 1 Departments of Neurological Surgery, University of California Davis, Davis, CA, USA; 2 Departments of Computer Science, University of California Davis, Davis, CA, USA; 3 Departments of Biomedical Engineering, University of California Davis, Davis, CA, USA; 4 Departments of Neurosurgery, Stanford University, Stanford, CA, USA; 5 Departments of Electrical Engineering, Stanford University, Stanford, CA, USA; 6 Departments of Mechanical Engineering, Stanford University, Stanford, CA, USA; 7 Departments of Computer Science, Stanford University, Stanford, CA, USA; 8 Wu Tsai Neurosciences Institute, Stanford University, Stanford, CA, USA; 9 Howard Hughes Medical Institute, Stanford University, Stanford, CA, USA; 10 Departments of Radiology and Neuroscience, Washington University School of Medicine, Saint Louis, MO, USA; 11 School of Engineering and Carney Institute for Brain Sciences, Brown University, Providence, RI, USA; 12 VA RR&D Center for Neurorestoration and Neurotechnology, VA Providence Healthcare, Providence, RI; 13 Center for Neurotechnology and Neurorecovery, Department of Neurology, Massachusetts General Hospital, Harvard Medical School, Boston, MA

## Abstract

Brain-computer interfaces can enable rapid, intuitive communication for people with paralysis by transforming the cortical activity associated with attempted speech into text on a computer screen. Despite recent advances, communication with brain-computer interfaces has been restricted by extensive training data requirements and inaccurate word output. A man in his 40’s with ALS with tetraparesis and severe dysarthria (ALSFRS-R = 23) was enrolled into the BrainGate2 clinical trial. He underwent surgical implantation of four microelectrode arrays into his left precentral gyrus, which recorded neural activity from 256 intracortical electrodes. We report a speech neuroprosthesis that decoded his neural activity as he attempted to speak in both prompted and unstructured conversational settings. Decoded words were displayed on a screen, then vocalized using text-to-speech software designed to sound like his pre-ALS voice. On the first day of system use, following 30 minutes of attempted speech training data, the neuroprosthesis achieved 99.6% accuracy with a 50-word vocabulary. On the second day, the size of the possible output vocabulary increased to 125,000 words, and, after 1.4 additional hours of training data, the neuroprosthesis achieved 90.2% accuracy. With further training data, the neuroprosthesis sustained 97.5% accuracy beyond eight months after surgical implantation. The participant has used the neuroprosthesis to communicate in self-paced conversations for over 248 hours. In an individual with ALS and severe dysarthria, an intracortical speech neuroprosthesis reached a level of performance suitable to restore naturalistic communication after a brief training period.

## Introduction:

Communication is a priority for the millions of people living with dysarthria from neurological disorders such as stroke and amyotrophic lateral sclerosis (ALS)^1^. People living with diseases that impair communication report increased rates of isolation, depression, and decreased quality of life^2,3^; losing communication often determines if a person will pursue or withdraw life-sustaining care in advanced ALS^4^. While existing augmentative and assistive communication technologies such as head or eye trackers are available, they suffer from low information transfer rates and become increasingly onerous to use as patients lose voluntary muscle control^5^. Brain-computer interfaces are a promising communication technology that can directly decode the user’s intended speech from cortical neural signals^6^. Efforts to develop a speech neuroprosthesis are built on largely offline (post hoc) studies using data from able-bodied speakers undergoing electrophysiological monitoring for clinical purposes (e.g. ^7–15^, but see ^16^). Several groups have performed online (real-time) brain-computer interface studies specifically to restore lost speech using chronically implanted electrocorticography (ECoG)^17–20^ and intracortical multielectrode arrays^21^. Two recent reports have established ‘brain-to-text’ speech performance^19,21^ by decoding the neural signals generated by attempted speech into phonemes (the building blocks of words), and assembling these phonemes into words and/or sentences displayed on a computer screen. These studies achieved communication performance, quantified using the word error rate metric, of 25.5% with a 1,024-word vocabulary^19^ and 23.8% with a 125,000-word vocabulary^21^, which is insufficient for reliable general-purpose communication. These prior studies also required at least 13 days of recording to collect sufficient training data to obtain that level of performance (17.7 training data hours in ^19^, 16.8 hours in^21^).

Here, we report an intracortical speech neuroprosthesis capable of providing access to a comprehensive 125,000-word vocabulary, with low training data requirements. We report that our clinical trial participant, living with advanced ALS and severe dysarthria, achieved very high accuracy brain-to-text communication (word error rates consistently below 5%), with useful function beginning on the very first day of use.

## Methods:

### Study participant

A left-handed man in his 40’s (referred to as ‘SP2’ in this preprint rather than the actual trial participant designation, which the participant is familiar with, as per medRxiv policy) with amyotrophic lateral sclerosis (ALS) was enrolled into the BrainGate2 pilot clinical trial. At the time of enrollment, he had no functional use of his upper and lower extremities and had severe dysarthria (ALSFRS-R = 23). For the duration of this study (8 months following implantation), he has maintained a modified mini-mental status exam score of 27 (the highest score attainable). At the time of this report, he still retains eye and neck movements, but has limited orofacial movement with a mixed upper- and lower-motor neuron dysarthria resulting in a monotone, low-volume, hypernasal speech with off-target articulation. He requires non-invasive respiratory support at night, and does not have a tracheostomy. When speaking with non-expert listeners, he is unintelligible (Audio 1): his oral motor tasks on the Frenchay Dysarthria Assessment-2 were an “E” rating, representing non-functioning or profound impairment. When speaking to expert listeners, he communicates at 6.8 ± 5.6 (mean ± standard deviation) correct words per minute. His typing speed using his gyroscopic headmouse (Zono 2, Quha, Nokia, Finland) is 6.3 ± 1.3 correct words per minute (Fig. S1). The severity of dysarthria has remained stable during the time-period of this report, including the immediate postoperative period. Additional participant details are in Section S1.01.

### Surgical implantation

The goal of the surgery was to implant four microelectrode arrays (NeuroPort Array, Blackrock Neurotech, Salt Lake City, Utah, USA) into the precentral gyrus, an important cortical target for coordinating the motor activities related to speech^17,19,21^. Each single electrode has one recording site ~50 μm in size, and is designed to record from a single or a small number of cortical neurons. A microelectrode array is 3.2 × 3.2 mm in size, has 64 recording sites in an 8 × 8 grid arrangement, and is inserted 1.5 mm into the cortex using a specialized high-speed pneumatic inserter.

We performed a left-sided 5 × 5 cm craniotomy under general anesthesia. Care was taken to avoid placing the microelectrode arrays through large vessels on the cortical surface, identified by visual inspection. Two arrays are connected to one percutaneous pedestal designed to transmit the neural recordings to external computers. We placed two percutaneous pedestals, for a total of 256 recording sites. Reference wires were placed in both the subdural and epidural spaces.

Our participant underwent surgery in July 2023, had no study-related serious adverse events, and was discharged on postoperative day 3. From initial incision to closure, the operation took 5 hours to perform. We began collecting data in August 2023, 25 days after surgery.

### Recording array implant locations and decoding contributions

**Figure 1.**
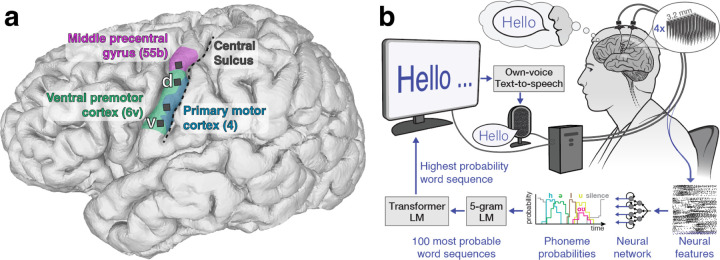
Electrode locations and speech decoding setup. **a**, Approximate microelectrode array locations, represented by black squares, superimposed on a 3d reconstruction of the participant’s brain. Colored regions correspond to the Human Connectome Project’s multi-modal atlas of cortical areas^22^ aligned to the participant’s brain using the Human Connectome Project’s MRI protocol scans before implantation, concordant with the precentral gyrus on a MNI template brain (Figure S11). **b**, Diagram of the brain-to-text speech neuroprosthesis. Cortical neural activity is measured from the left ventral precentral gyrus using four 64-electrode Utah arrays. Machine learning techniques decode the cortical neural activity into an English phoneme every 80 ms. Using a series of language models (LM), the predicted phoneme sequence is translated into a series of words that appear on a screen as the participant tries to speak. At the end of a sentence, an own-voice text-to-speech algorithm vocalizes the decoded sentence designed to emulate the participant’s voice prior to developing ALS (Section S5).

Prior to implanting arrays in the precentral gyrus, we identified the central sulcus on an anatomical MRI scan, and confirmed that our study participant was left-hemisphere language dominant on functional MRI, using standard clinical fMRI tasks (sentence completion, silent word generation, silent verb generation, and object naming). In addition to pre-operative morphological MRI and fMRI studies, we further refined our implantation targets using the Human Connectome Project’s multi-modal MRI-derived cortical parcellation precisely mapped to the participant’s brain^22^ (Fig. 1a, Fig. S2, Section S1.02). We targeted language-related area 55b^23^ and three areas in the precentral gyrus associated with motor control of the speech articulators: premotor areas dorsal 6v (d6v) and ventral 6v (v6v), and primary motor cortex (area 4; Fig. S2). Our choice of targeting speech motor cortex was motivated by our previous study, which found that two 6v arrays provided informative signals for speech decoding^21^. Compared to that previous report, the implantation of four micro-electrode arrays doubles the number of electrodes implanted into the ventral precentral gyrus and doubles the corresponding cortical area covered.

### Real-time acquisition and processing of neural data

Neural activity was transmitted from the pedestals to a computer system designed to decode the activity in real-time (Fig. 1b). A signal processing system (NeuroPort System, Blackrock Neurotech) was used to acquire signals from the two pedestals (Fig. S3) and send them to a series of commercially available computers running custom software^24^ (Section S1.5) for real-time signal processing (Section S1.4) and decoding (Sections S2, S3). The system sits on a wheeled cart and requires the computers to be connected to standard wall outlets. Blackrock Neurotech was not involved in the data collection or reporting in this study, and had no oversight regarding the decision to publish.

### Speech task designs

The study consisted of 84 data collection sessions over the course of 32 weeks (Section S1.06; Table S2) and took place in the participant’s home. Each session consisted of a series of task blocks, lasting approximately 5–30 minutes, wherein the participant used the neuroprosthesis. Between blocks, he would take breaks, eat meals, etc. One session was performed on any day. During each block, the participant used the system in two different ways: 1) an instructed-delay Copy Task (Videos 1, 2 and Section S1.07); and 2) a self-paced Conversation Mode (Videos 4, 5 and Section S1.08). The instructed-delay task consisted of words being presented on a computer screen, and the participant attempted to say the words after a visual/audio cue^21^. The self-paced Conversation Mode involved the participant trying to say whatever he wanted, from the 125,000 word dictionary, in an unstructured conversational setting. In both tasks, speech decoding occurred in real-time: as he spoke, the cortical activity at the four micro-electrode arrays were recorded and interpreted, and the predicted words were presented on the screen. Completed sentences were read aloud by a computer program and, in later sessions, automatically punctuated (Sections S4 and S3.03). The neuroprosthesis could also send the sentence to the participant’s personal computer by acting as a bluetooth keyboard, which allowed him to use it for activities such as writing emails (Section S1.08). Sampled phoneme and words used for decoder training accumulated over the course of the study (Figure S4).

### Decoding speech

Our neuroprosthesis was designed to translate the participant’s cortical neural activity into words when he attempted to speak (Section S2, Figs. S5, S6). No microphone input was used for decoding, and we found no evidence of acoustic or vibration-related contamination in the recorded neural signals (Section S4.03, Fig. S7). Every 80 ms, the activity from the population of cortical neurons was used to predict the most likely English phoneme (the building block of sounds of a language) being attempted. Phoneme sequences were then combined into words using an openly-available language model^21^. Next, we applied two further open-source language models to translate the sequence of words into the most likely English sentence. Hence, the neural activity was the only input for all subsequent language-model refinements (Section S3, Fig. S8), as described in ^21^. Machine learning techniques enabled high accuracy decoding; data from different days were combined to continuously calibrate the decoder, enabling maintenance of high performance during neuroprosthesis use (Section S2 and Figs. S9, S10).

### Evaluation

We used two measures to analyze the speech decoding performance: phoneme error rate and word error rate, consistent with previous speech decoding studies^17,19,21^. These measures are the ratio of phonemic or word errors to the total number of phonemes or words expected to be decoded, respectively. An error is defined as when an insertion, deletion, or substitution is needed to have the decoded sentence match the intended sentence. The phoneme error rate can be understood as the system’s ability to translate cortical neural activity into phonemes without language models (to make this explicit, we label it as ‘raw’ phoneme error rate in figures), and word error rate as an estimate of overall communication accuracy. Data was collected in continuous blocks (lasting 5–30 minutes in length) which were separated by short breaks. Blocks were either “training blocks” in which data were collected for decoder training or optimization, or predetermined “evaluation blocks” used to measure and report neuroprosthesis performance. Reported error rates are aggregated over all evaluation sentences for each session (Section S1.09). The first-ever closed-loop block (session 1) was excluded from evaluation because the participant cried with joy as the words he was trying to say correctly appeared on-screen; the research team paused the evaluation until after the participant and his family had a chance to celebrate the moment.

### Statistical analyses

Results for each analysis are presented with 95% confidence intervals or as mean ± standard deviation. Confidence intervals were estimated by randomly resampling each dataset 10,000 times with replacement, and have not been adjusted for multiplicity. The evaluation metrics for decoding performance (phoneme error rate and word error rate) were chosen before the start of data collection (Section S1.09).

## Results:

### Online decoding performance

**Figure 2.**
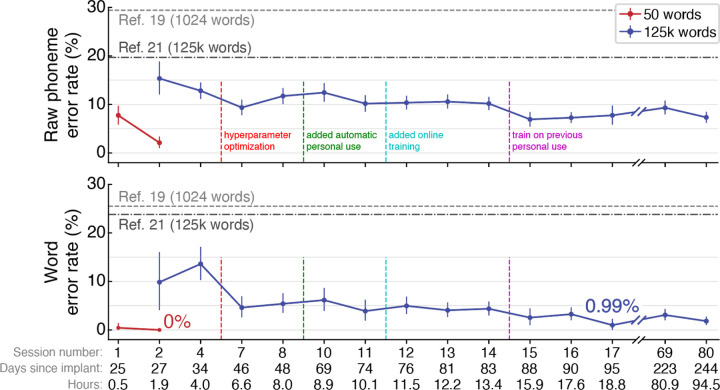
Online speech decoding performance. Phoneme error rates (top) and word error rates (bottom) are shown for each session for two vocabulary sizes (50 versus 125,000 words). Reference error rates are plotted (horizontal dashed lines) for two previous speech neuroprosthesis studies^19,21^. The horizontal axis displays the research session number, the number of days since arrays implant, and the cumulative hours of neural data used to train the speech decoder for that session. Aggregate error rates across all evaluation sentences are shown for each session (mean ± 95% confidence interval). Vertical dashed lines represent when decoder improvements were introduced. Fig. S20 shows phoneme and word error rates for individual blocks.

In the first session, the participant attempted to speak prompted sentences constructed from a 50-word vocabulary^17^. We recorded 213 sentences (30 minutes) of Copy Task data, which were used to calibrate the speech neuroprosthesis. Next, we decoded his neural cortical activity in real-time as he tried to speak. The neuroprosthesis decoded his attempted speech with a word error rate of 0.44% (95% confidence interval [CI], 0.0% to 1.4%). We replicated this result for 50-word vocabulary decoding in the second research session, in which all of the participant’s attempted sentences were decoded correctly (0% word error rate; Fig. 2).

In this second research session, we also expanded the vocabulary of the neuroprosthesis from 50 words to over 125,000 words, which encompasses the majority of the English language^25^. We collected an additional 260 sentences of training data (1.4 hours). After being trained on these additional data, the neuroprosthesis decoded the participant’s attempted speech with a word error rate of 9.8% (95% CI, 4.1% to 16.0%; Fig. 2); offline analyses suggest that even better first-session performance may have been possible given the information content of the recorded neural signals (Figs. S12, S13). Performance continued to improve in subsequent research sessions as we collected more training data and adapted innovations for incorporating new data more effectively^26^. The neuroprosthesis achieved a word error rate of 2.5% (95% CI, 1.0% to 4.5%) by session 15, and high accuracy decoding performance was maintained through session 84, more than eight months after implant. Average Copy Task decoding performance in the final 5 evaluation sessions had a word error rate of 2.5% (95% CI, 2.0% to 3.1%) at the participant’s self-paced speaking rate of 31.6 words per minute (95% CI, 31.2% to 32.0%; Fig. S1), with individual day’s average word error rates ranging from 1.0% to 3.3% (Table S3). The neuroprosthesis’ communication rate far exceeded the participant’s standard means of communication using a head mouse or skilled interpreter (Fig. S1a).

The system achieved high accuracy at the start of sessions, maintaining an average word error rate of 4.9% (95% CI, 3.7% to 6.2%) over the first 50 sentences of seven sessions (Fig. S14). Offline analyses indicated that this rapidly-available communication capability was enabled by the neuroprosthesis being able to maintain accurate decoding for at least twenty days without any new training data, and for multiple months when continuously fine-tuning the neuroprosthesis^26^ (Fig. S15). In decreasing order, the most informative electrode arrays for decoding speech were in areas ventral 6v (best), 55b, 4, and dorsal 6v (worst; Fig. S16). Decoding errors tended to occur between phonemes that are articulated similarly (Fig. S17). The speech decoder generalized to new words, and accuracy improved for a given word the more often it appeared in the training data (Fig. S18). It could also generalize to different attempted amplitudes of speech, including non-vocalized speech (Fig. S19).

### Conversational speech using the brain-computer interface

**Figure 3.**
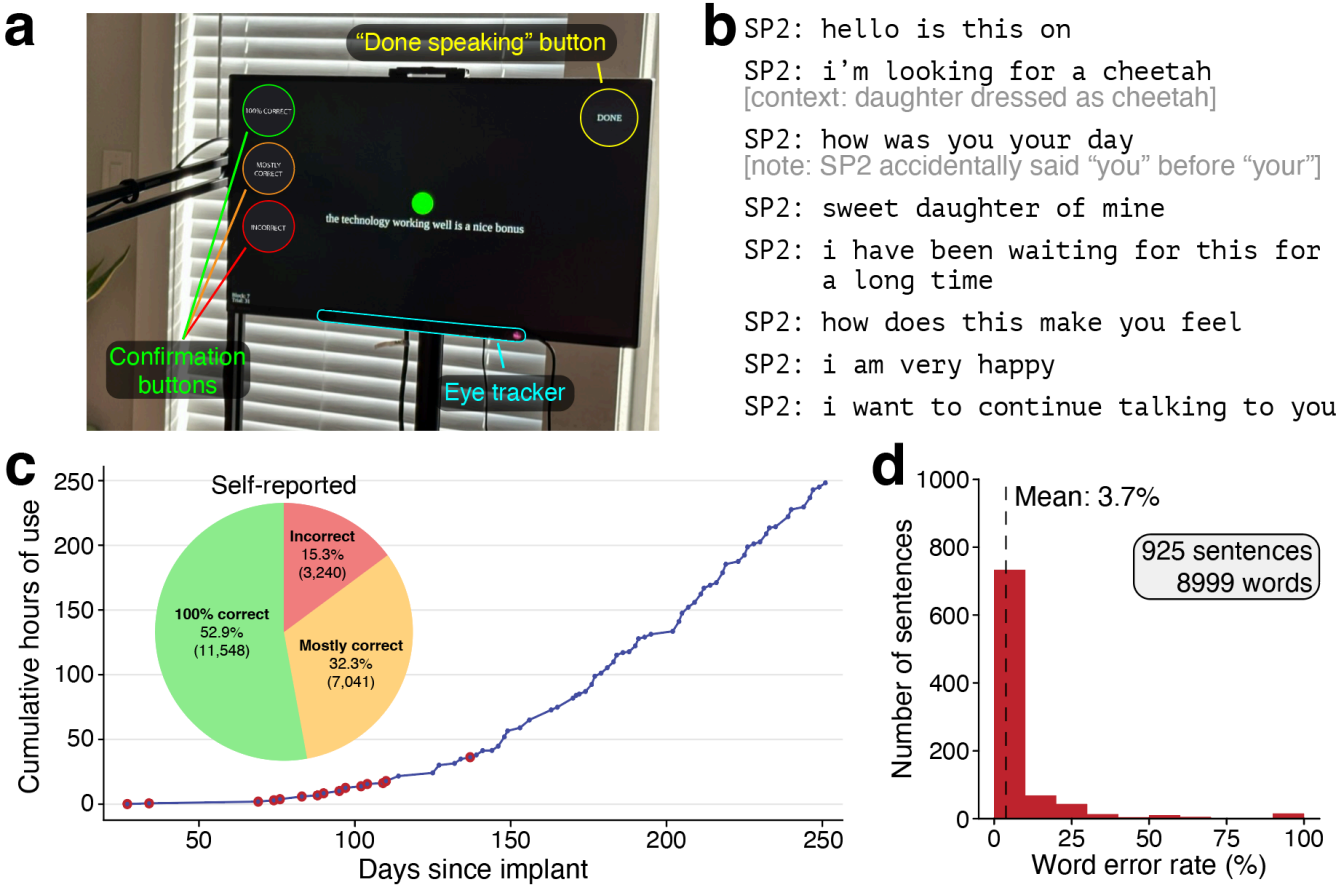
Extensive use of the neuroprosthesis for accurate self-initiated speech. **a**, Photograph of the participant and speech neuroprosthesis in Conversation Mode. The neuroprosthesis detected when he was trying to speak solely based on neural activity, and concluded either after 6 seconds of speech inactivity, or upon his optional activation of an on-screen button via eye tracking. After the decoded sentence was finalized, the participant used the on-screen confirmation buttons to indicate if the decoded sentence was correct. **b**, Sample transcript of our participant using the speech neuroprosthesis to speak to his daughter on the second day of use (Video 3). Additional transcripts are available in Table S4. **c,** Cumulative hours that the participant used the speech neuroprosthesis to communicate with those around him in structured research sessions and during personal use. For sessions represented by points outlined in red, decoding accuracy is quantified in (**d**). The distribution of self-reported decoding accuracy for each sentence across all Conversation Mode data (n = 21,829) is shown in the inset pie chart. Sentences where the participant did not self-report decoding accuracy within 30 seconds of sentence completion are excluded (n = 868). **d**, Evaluating speech decoding accuracy in conversations (n = 925 sentences with known true labels, sourced from red-labeled sessions in (**c**)). The average word error rate was 3.7% (95% CI, 3.3% to 4.3%).

We developed a system for the participant to have conversations via self-initiated speech. Relying exclusively on cortical neural activity, the speech neuroprosthesis detected when he started or stopped speaking and decoded his attempted speech accordingly (Section S1.08, Fig. S21). The system rarely falsely detected that he wanted to speak (Fig. S22). Additionally, the participant had the option to use an eye tracker for selecting actions (Fig. 3a) to i.) finalize and read aloud the sentence, ii.) indicate whether the sentence was decoded correctly or not, or iii.) spell out words letter-by-letter that were not correctly predicted by the decoder (e.g., because they were not in the vocabulary, such as certain proper nouns).

The participant’s first use of the neuroprosthesis for naturalistic communication with his family is exemplified in Fig. 3b (Video 3; Table S4 provides additional transcripts). In subsequent sessions, he utilized the neuroprosthesis for personal use (e.g., Videos 4–5), communicating a total of 1189 sentences from sessions 1–31. For the majority of these Conversation Mode sentences (925 sentences; 77.8%) we were able to confirm the participant’s intended speech through directly asking him, contextual analysis, and examining the decoded phoneme probability patterns. Self-initiated sentences for which we knew the ground-truth were decoded with a word error rate of 3.7% (95% CI, 3.3% to 4.3%; Fig. 3d), although we note that the word error rate reported only for sentences with known ground truth labels may be inflated (Section S1.09). For one session where we validated the ground truth of every sentence (43 sentences, 873 words) with the participant, the word error rate was 2.5% (95% CI, 1.3% to 4.0%). Using the neuroprosthesis, the participant told the research team, “I hope that we are very close to the time when everyone who is in a position like me has the same option to have this device as I do” (Table S4). The participant used the neuroprosthesis in this Conversation Mode during 72 (out of 84 total) sessions over 8 months (248.3 cumulative hours; 22,697 sentences). The longest continuous use of the speech neuroprosthesis in Conversation Mode was 7.7 hours. He used it to perform activities ranging from talking to the research team and his family and friends, to performing his occupation by participating in videoconferencing meetings and writing documents and emails.

## Discussion:

Beginning on the first day of device use, a brain-to-text speech neuroprosthesis with 256 recording sites in the left precentral gyrus accurately decoded intended speech in a man with severe dysarthria due to ALS. He communicated using a comprehensive 125,000 word vocabulary on the second day of use. Within 16 hours of use, the neuroprosthesis correctly identified 97.3% of attempted words. To contextualize this 2.7% word error rate, the state-of-the-art for English automated speech recognition (e.g., smartphone dictation) has an approximate 5% word error rate^27^ and able speakers have a 1–2% word error rate^28^ when reading a paragraph aloud. We believe that the high decoding accuracy demonstrated in this study indicates that speech neuroprostheses have reached a level of performance suitable for rapidly and accurately restoring communication to people living with paralysis.

This study’s participant used the brain-to-text speech neuroprosthesis to converse with family, friends, healthcare professionals, and colleagues. His regular means of communication without a neuroprosthesis involved either (1) having expert caregivers interpret his severely dysarthric speech, or (2) using a head-mouse with point-and-click selections on a computer screen. The investigational BrainGate Neural Interface System became his preferred way to communicate with our research team, and he used it on his own time (with a researcher’s assistance to connect and launch the system) to be able to more rapidly write and communicate as part of his occupation and family life. The participant and his family and friends also reported being pleased with the own-voice text-to-speech at the end of each sentence; they indicated that the system’s voice did resemble his own.

This study demonstrated a large reduction in the quantity of training data required to achieve high accuracy decoding. In our previous study^21^, the participant attempted to speak 260–480 sentences at the start of each day, after which up to ~30 minutes of computation time was required until the speech neuroprosthesis was ready for use. That previous study’s reported closed-loop results were measured starting 113 days post-implant, and used 16.8 hours of training data, collected over 15 days, to achieve a word error rate of 23.8%. A previous ECoG speech neuroprosthesis required 17.7 hours of training data, collected over 13 days, to reach a word error rate of 25.5%^19^. The new neuroprosthesis demonstrated here provided over 99% accuracy on a limited set of 50 words^17^ after just 30 minutes of training data on the very first day of use. It also achieved over 95% accuracy on a large vocabulary after collecting 6.6 cumulative hours of training data (over 7 sessions), and offline analyses indicate that optimized methods could provide >91% accurate large-vocabulary communication on the first day of use.

Previous studies have reported that intracortical devices require frequent recalibration^29–31^. Motivated by recent studies showing that multiple days of neural data can be used to calibrate an effective decoder for a new day^32,33^, here we demonstrated that a speech decoder trained with multiple previous days of data could similarly be used to provide >95% accuracy at the start of a new session. Future work is needed to establish whether the strategy we employed^26^ can maintain performance indefinitely in the absence of ground-truth labels of intended speech, but it is encouraging that across the 29 session days where the participant used the neuroprosthesis solely for personal use, he only requested to recalibrate the decoder on three occasions. Each calibration took approximately 7.5 minutes, during which twenty Copy Task sentences were displayed to provide ground-truth training labels and thereby rapidly update the decoder.

It is possible that an important factor enabling the higher performance of this study relative to our prior intracortical speech neuroprosthesis^21^ was doubling the number of microelectrodes in speech motor cortex. Our finding that ~200 electrodes in these regions is sufficient for very high accuracy brain-to-text communication provides an important design parameter to guide ongoing efforts to build neural interface hardware that can reach patients at scale. Using an improved phoneme-to-sentences language model (relative to ^21^) also improved performance (Fig. S8), and the participant’s slow speaking rate (Fig. S1) may also have contributed.

In addition to recording from two arrays in the putative ventral portion of area 6v (speech motor cortex) as in ^21^, we also targeted one array each into two areas which, to our knowledge, have not previously been recorded from with multielectrode arrays: area 4 (primary motor cortex, which in humans is often in the sulcus^22^ and thus largely not accessible with Utah arrays) and area 55b. We found that the strongest phoneme encoding was from the array in ventral 6v, which is consistent with our previous participant^21^. The array in area 4 also showed high phoneme encoding, as did the array in area 55b, which has recently been proposed as an important node in the wider speech production network^23^. We note that these brain area descriptions are estimations based on precisely aligning the participant’s brain to a Human Connectome Project derived atlas using multi-modal MRI.

## Limitations:

As with other recent clinical trial reports in the nascent field of implanted speech brain-computer interfaces^17–19,21^, this study involved a single participant. Future work with additional participants is needed to establish the across-individual distribution of performances for speech decoding. Whether similar results can be expected in future users may depend on whether the signal-to-noise ratio of this participant’s speech-related neural signal modulation is typical. Nevertheless, these data, when combined with our previous speech decoding results with two 64-electrode arrays in area 6v^21^, demonstrate both successful initial replication and subsequent methodological improvements of the intracortical speech neuroprosthesis approach. Furthermore, while the demonstrated brain-to-text capabilities can provide widely useful communication, they do not capture the full expressive richness of voice; the more difficult challenge of closed-loop brain-to-voice synthesis remains an active area of speech neuroprosthesis research^19,34^. Notable steps that remain before brain-to-text neuroprostheses are likely to be adopted at scale include removing the percutaneous connector (i.e., making the electrodes fully implanted with wireless power and telemetry), reducing the size and number of computers used for processing, and automating the software so that users and their care partners can operate it independently.

### Decoding speech in patients with other neurological impairments

The participants in both this study and our previous report^21^ had dysarthria due to ALS. Further work is needed to assess whether similar methods will work for other etiologies of dysarthria. Given that we recorded from ventral precentral gyrus, which is upstream of the neuronal injury incurred in many conditions, and that recent ECoG speech neuroprostheses were demonstrated in two individuals with brainstem stroke^17–19^, we predict that this approach will also work in other conditions^35^.

We note that the participant retains voluntary (albeit impaired) bulbar muscle control, normal sensation, and normal hearing. We do not know whether our approach will continue to restore communication should he develop anarthria. We also do not know if our approach will work in patients who already have anarthria or have hearing impairments.

### Long-term stability of neural decoding

Prior reports have described that neural recording quality can decrease over years with the type of microelectrode array used in this study (e.g., ^36^). While intracortical neural decoding of attempted hand movements has been shown to be sustained beyond five years^37–40^, our study reports data only up to 8 months after implantation. It is not known how well decoding performance will be sustained over time.

## Conclusion:

Overall, the rapid and highly accurate restoration of full vocabulary, speech-based communication, enabled by an intracortical neuroprosthesis and used by a person with advanced ALS, suggests that this approach may be useful in improving the communication ability and autonomy of people with severe speech and monitor impairments.

## Supplementary Material

Supplement 1
